# Hypoxia-Inducible Factor-1α Activity as a Switch for Glioblastoma Responsiveness to Temozolomide

**DOI:** 10.3389/fonc.2018.00249

**Published:** 2018-07-02

**Authors:** Alessia Lo Dico, Cristina Martelli, Cecilia Diceglie, Giovanni Lucignani, Luisa Ottobrini

**Affiliations:** ^1^Institute of Molecular Bioimaging and Physiology (IBFM), CNR, Milan, Italy; ^2^Department of Pathophysiology and Transplantation, University of Milan, Milan, Italy; ^3^Department of Health Sciences, University of Milan, Milan, Italy; ^4^Department of Diagnostic Services, Unit of Nuclear Medicine, San Paolo Hospital, Milan, Italy

**Keywords:** theranostic biomarker, hypoxia-inducible factor-1α silencing, apoptosis, chaperone-mediated autophagy activity, temozolomide responsiveness

## Abstract

**Rationale:**

The activity of the transcription factor, hypoxia-inducible factor (HIF)-1α, is a common driver of a number of the pathways involved in the aggressiveness of glioblastomas (GBMs), and it has been suggested that the reduction in this activity observed, soon after the administration of temozolomide (TMZ), can be a biomarker of an early response in GBM models. As HIF-1α is a tightly regulated protein, studying the processes involved in its downregulation could shed new light on the mechanisms underlying GBM sensitivity or resistance to TMZ.

**Methods:**

The effect of HIF-1α silencing on cell responsiveness to TMZ was assessed in four genetically different human GBM cell lines by evaluating cell viability and apoptosis-related gene balance. LAMP-2A silencing was used to evaluate the contribution of chaperone-mediated autophagy (CMA) to the modulation of HIF-1α activity in TMZ-sensitive and TMZ-resistant cells.

**Results:**

The results showed that HIF-1α but not HIF-2α activity is associated with GBM responsiveness to TMZ: its downregulation improves the response of TMZ-resistant cells, while blocking CMA-mediated HIF-1α degradation induces resistance to TMZ in TMZ-sensitive cells. These findings are in line with the modulation of crucial apoptosis-related genes.

**Conclusion:**

Our results demonstrate the central role played by HIF-1α activity in determining the sensitivity or resistance of GBMs to TMZ, and we suggest that CMA is the cellular mechanism responsible for modulating this activity after TMZ treatment.

## Introduction

Glioblastomas (GBMs) are the most frequently encountered astrocyte-derived brain tumors in adults and are characterized by highly aggressive behavior that leads to a poor prognosis (1). Despite the multimodal treatment approaches based on temozolomide (TMZ) chemotherapy and radiotherapy, the median survival of patients with GBM is approximately 14.6 months, and their 5-year survival rate is 4–5% (2).

Temozolomide exerts its anti-tumor action by methylating the O6 position of guanine residues, and these *O*-6-methylguanine (*O*-6-MG) adducts play a critical role in its activity by blocking gene transcription, thus increasing GBM sensitivity to radiation and activating pro-autophagic and apoptotic processes (3, 4). Moreover, a functional relationship between apoptosis and autophagy in tumorigenesis has also been described in relation to TMZ responses (4, 5).

Drug resistance is a major issue in the management of GBMs (6). It is now widely accepted that the methylation of the *O*-6-methylguanine DNA methyltransferase (MGMT) promoter is a biomarker of TMZ response as the enzyme antagonizes the effect of alkylating agents, thus counteracting their cytotoxicity and acting as a negative prognostic factor (7–9). However, there are other biomarkers that are capable of estimating the efficacy of TMZ (10–12): for example, we have proposed hypoxia-inducible factor (HIF)-1α activity as an early biomarker of response to TMZ in U251 GBM cells (13).

Hypoxia-inducible factor is related to a number of the characteristics of GBMs involved in tumor progression, malignancy, and treatment resistance (14–16). It not only drives the expression of the genes involved in the response to hypoxia but also regulates the expression of genes that play a major role in other intracellular processes such as invasion, epithelial–mesenchymal transition, metastasis, dedifferentiation and maintenance of cancer stem cells, and genomic instability (17, 18). Recent studies (19, 20) have shown that HIF-1α downregulation reduces MGMT expression in GBM stem cells, thus suggesting a possible correlation between the two proteins (11, 19, 21–25), and we have previously shown that the negative modulation of HIF-1α is related to the response of U251 GBM cells to TMZ *in vitro* and *in vivo* (13).

The aim of this study was to investigate the role of HIF-1α activity as a biomarker of responsiveness to TMZ in a panel of glioma cell lines characterized by different MGMT methylation status and genetic background.

Hypoxia-inducible factor-1α degradation is due to the balance between the activity of the proteasome and chaperone-mediated autophagy (CMA) machinery (3, 26). CMA is a form of selective autophagy involved in the degradation of proteins containing a specific KFERQ-like motif. Different chaperone proteins such as Hsp90, STUB/CHIP, and Hsc70 cooperate in shuttling target proteins to specific lysosomes where, after LAMP-2A binding and multimerization, the KFERQ-containing proteins are unfolded and degraded. CMA regulation is mainly based on LAMP-2A localization and multimerization (27). As previous data show that TMZ induces autophagy (28, 29) and reduces HIF-1α activity (13) and that HIF-1α is a CMA target (30), we also investigated the role of CMA in modulating HIF-1α activity and, consequently, determining responsiveness or resistance to TMZ treatment.

Finally, the results of this study allow us to propose HIF-1α activity as an important therapeutic target in GBMs and to consider it as a theranostic biomarker (10, 31–33).

## Results

### TMZ Dose–Response Study Confirmed the Different Sensitivity to Treatment of the Selected Cell Lines

**Figure 1:**
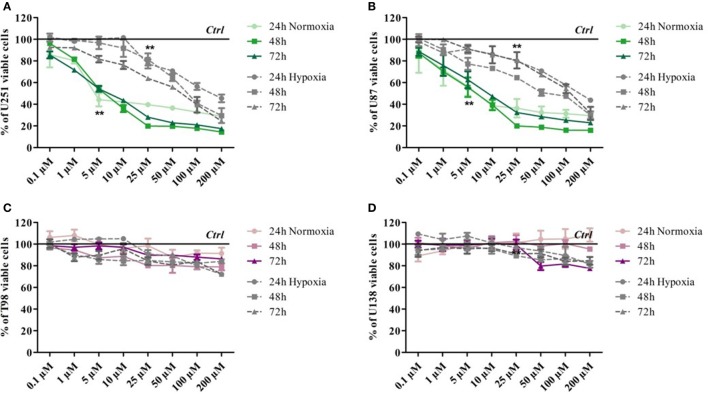
Dose–response viability of responsive and resistant cells after temozolomide (TMZ) treatment. Cell viability was assessed by means of a Trypan blue exclusion test and expressed as the percentage of viable cells after 24, 48, or 72 h of treatment at increasing doses of TMZ under normoxic and hypoxic conditions. **(A)** U251, **(B)** U87, **(C)** T98, and **(D)** U138. **p < 0.01 TMZ-treated cells vs untreated cells. Mean values ± SD.

Glioma cell lines showed different sensitivity to TMZ according to their genetic background. Cell viability was determined by means of a Trypan blue exclusion test under normoxic and hypoxic conditions after exposure to increasing doses of TMZ for 24, 48, and 72 h. Under normoxic conditions, the U251 and U87 cells showed dose-dependent responsiveness to TMZ treatment at each time point, even at very low drug concentrations (Figures 1A,B), whereas the T98 and U138 cells did not show any reduction in viability in response to treatment, thus confirming their TMZ resistance (Figures 1C,D). Under hypoxic conditions, the sensitivity of the responsive cells to TMZ was reduced: the TMZ dose required to induce a statistically significant reduction in cell viability was five times higher than under normoxic conditions (Figures 1A,B), whereas there was no change in the responsiveness of the TMZ-resistant cells (Figures 1C,D).

### HIF Activity as Biomarker of Treatment Efficacy in Glioma Cells

As previous data from our group suggest that HIF activity may be biomarker of TMZ treatment efficacy in U251 cells (13), HIF-1α expression and its nuclear translocation were investigated in all of the cell lines in relation to TMZ treatment and normoxic/hypoxic conditions. HIF-1α activity was also monitored in the same cells by assessing the expression of the vascular endothelial growth factor (VEGF) transcript (an HIF target gene) and its extracellular release.

**Figure 2:**
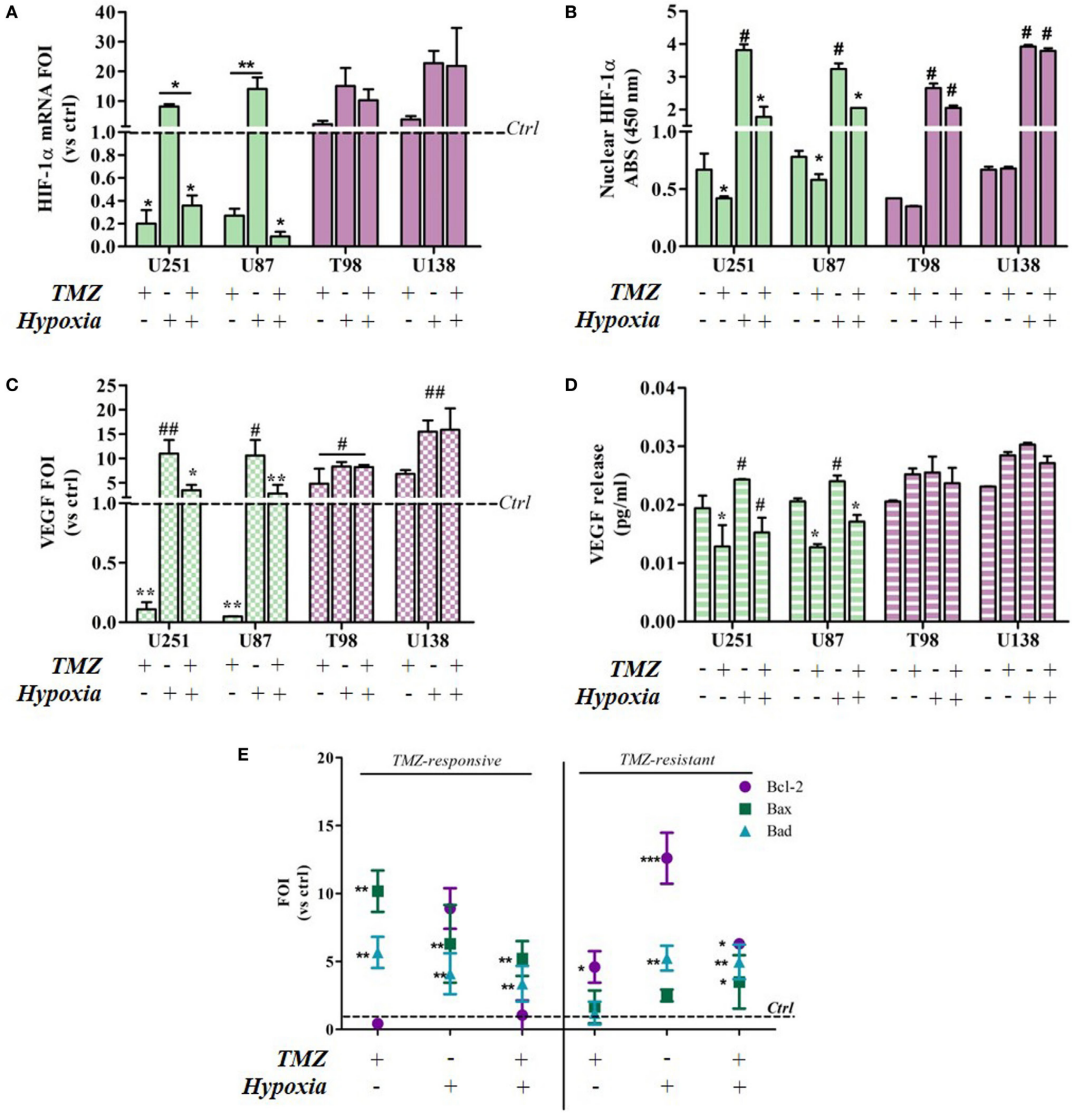
Temozolomide (TMZ) modulation of hypoxia-inducible factor (HIF)-1α expression and activity and apoptotic gene expression profiles in TMZ-sensitive and TMZ-resistant glioblastoma cells. **(A)** HIF-1α expression revealed by real-time PCR after 24 h treatment with TMZ 100 µM under normoxic and hypoxic conditions. The data were normalized to β-actin, and the ΔΔCt values were expressed as folds of induction (FOI). *p < 0.05; **p < 0.01 treated vs untreated cells. **(B)** ELISA-based HIF-1α nuclear quantification after TMZ treatment under normoxic and hypoxic conditions. The data are expressed as absorbance at 450 nm. *p < 0.05 vs control under normoxic conditions; #*p* < 0.05 vs control under hypoxic conditions. **(C)** Vascular endothelial growth factor (VEGF) expression revealed by real-time PCR after treatment with TMZ 100 µM under normoxic and hypoxic conditions. The data were normalized as above. *p < 0.05; **p < 0.01 TMZ-treated vs control cells under normoxic conditions; ^#^*p* < 0.05, ^##^*p* < 0.01 TMZ-treated vs control cells under hypoxic conditions. **(D)** ELISA of the VEGF released by glioma cells in cell medium after TMZ treatment. *p < 0.05 TMZ-treated vs control cells under normoxic conditions; ^#^*p* < 0.05 TMZ-treated vs control cells under hypoxic conditions. **(E)** The induction of pro-apoptotic (*Bad* and *Bax*) and anti-apoptotic genes (*Bcl-2*) was analyzed by means of real-time PCR in glioma cells treated with TMZ under normoxic and hypoxic conditions. The data were normalized to β-actin, and the ΔΔCt values were expressed as the ratio between the mean values in the responsive and resistant cells (FOI). *p < 0.05; **p < 0.01; ***p < 0.001 treated vs control cells. Mean values ± SD of three independent experiments.

Hypoxia-inducible factor-1α expression decreased after TMZ treatment in the responsive U251 and U87 cells, but not in the resistant cells (Figure 2A). As expected, hypoxia induced an increase in HIF-1α expression in all of the cell lines. Under both conditions, TMZ treatment significantly reduced HIF-1α transcription only in the responsive cells, and the HIF-1α nuclear localization assay confirmed these observations (Figure 2B). In fact only in the TMZ-resistant T98 and U138 cells, nuclear HIF-1α levels, even if increased in hypoxic conditions, were unaffected by TMZ treatment in both settings (Figure 2B). The modulation of HIF-1α activity was confirmed by the assessment of VEGF expression and release (Figures 2C,D). VEGF expression and release were positively modulated by hypoxia and negatively regulated by TMZ treatment only in the responsive cells, whereas only hypoxia modified VEGF levels in resistant cells.

The molecular consequences of the above culture conditions and treatments were analyzed by assessing apoptosis-related gene expression levels (*Bax, Bad*, and *Bcl-2*). Under normoxic conditions, TMZ treatment induced a reciprocal *Bax/Bad* vs *Bcl-2* balance in sensitive and resistant cells, and their expression patterns were similar to those induced by hypoxia. Interestingly, the hypoxia-induced expression pattern was reverted by TMZ treatment only in the sensitive cells (Figure 2E). There was no difference in gene expression levels in the four cell lines under control conditions (Figure S1 in Supplementary Material).

### HIF-1α but Not HIF-2α Impairment Was Associated With TMZ

**Figure 3:**
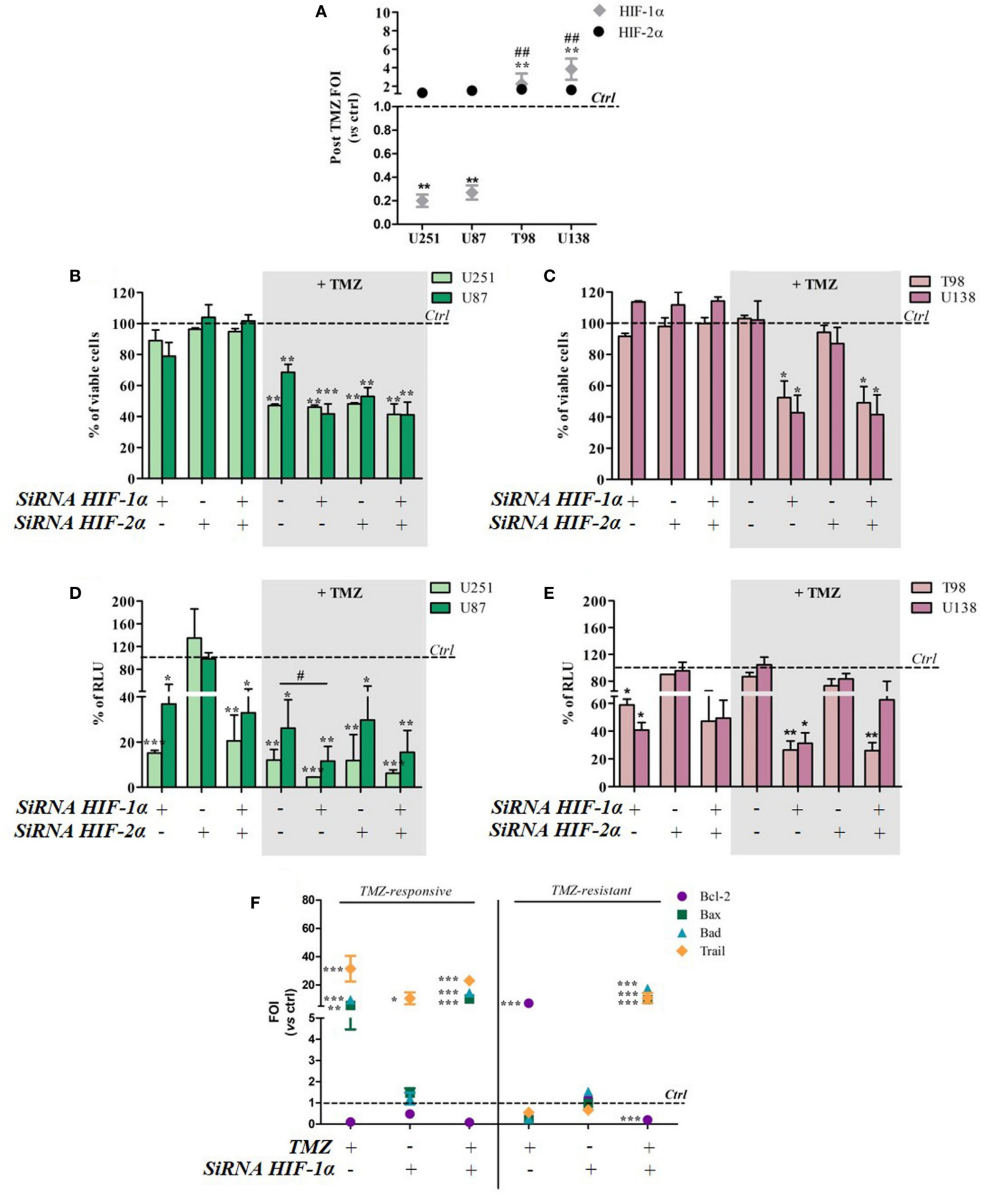
Hypoxia-inducible factor (HIF)-1α silencing induces a responsive-profile in temozolomide (TMZ)-resistant cells. **(A)** The expression of HIF-1α and HIF-2α was evaluated by means of real-time PCR after TMZ treatment in all of the cell lines. The data were normalized to β-actin, and the ΔΔCt values were expressed as folds of induction (FOI) **p < 0.01 treated vs control cells. ^##^*p* < 0.01 HIF-1α in resistant vs sensitive cells. **(B,C)** The viability of U251 and U87 **(B)** and T98 and U138 cells **(C)** was assessed by means of Trypan blue exclusion test, and expressed as the percentage of viable cells after HIF-1α or HIF-2α silencing. *p < 0.05, **p < 0.01, ***p < 0.001 vs control cells. **(D,E)** HIF-dependent luciferase activity was analyzed in cell lysates, and expressed as the percentage variation in relative luminescence units (RLUs). *p < 0.05; **p < 0.01; ***p < 0.001 vs control cells; ^#^*p* < 0.05 siRNA + TMZ vs TMZ. **(F)** After silencing, the cells were treated with TMZ and the induction of *Bax, Bad, Trail*, and *Bcl-2* genes was analyzed by means of real-time PCR. The data were normalized to β-actin, and the ΔΔCt values were expressed as FOI of the ratio between treated and control cells. Results of sensitive and resistant cells were presented as FOI mean values ± SD. *p < 0.05; **p < 0.01; ***p < 0.001 treated vs control cells. Mean values ± SD of three independent experiments.

To investigate whether TMZ can modulate HIF-1α and HIF-2α activity and expression, the two transcription factors were individually or concomitantly silenced in TMZ-sensitive and TMZ-resistant GBM cells. TMZ treatment did not modulate HIF-2α expression in either cell type, but it downregulated HIF-1α expression in sensitive cells and upregulated it in resistant cells (Figure 3A). The changes were independent of basal expression levels (Figure S2 in Supplementary Material).

Viability tests carried out after TMZ treatment and/or HIF-1α or HIF-2α silencing showed that the silencing of one or both HIF transcription factors alone did not reduce cell viability in any of the cell lines. After TMZ treatment, HIF-1α silencing did not lead to any further cell mortality beyond that obtained with TMZ treatment alone in the responsive U251 and U87 cell lines (Figure 3B). Otherwise, HIF-1α (but not HIF-2α) silencing significantly decreased post-treatment cell viability in the resistant T98 and U138 cells (Figure 3C).

As expected, biochemical luciferase assay monitoring showed that HIF activity was reduced by TMZ only in sensitive cells, and further decreased after HIF-1α (but not HIF-2α) silencing. TMZ treatment alone did not reduce HIF-1α activity in resistant cells that was significantly reduced only in combination with HIF-1α silencing (Figures 3D,E). Silencing efficiency is shown in Figure S3 in Supplementary Material, which also shows that, under these conditions, luciferase expression and activity reflects HIF-1α but not HIF-2α activity.

The molecular consequences of HIF-1α silencing were evaluated by monitoring the expression of the apoptosis-related genes *Bax, Bad, Trail*, and *Bcl-2*. Again, TMZ induced an inverse modulation of pro- and anti-apoptotic genes in sensitive and resistant cells. However, HIF-1α silencing in sensitive cells did not induce any further modulation of apoptosis-related genes beyond that obtained after TMZ treatment but, interestingly, HIF-1α silencing in resistant cells restored a “sensitive-like pattern” in apoptosis-related gene expression, with the upregulation of *Bax, Bad*, and *Trail* expression, and a reduction in *Bcl2* transcript levels (Figure 3F).

Temozolomide efficacy was also monitored after pharmacological HIF-1α activity impairment using BEZ235 and LY294002 (PI3K/AKT inhibitors) and Trametinib (MAPK inhibitor).

**Figure 4:**
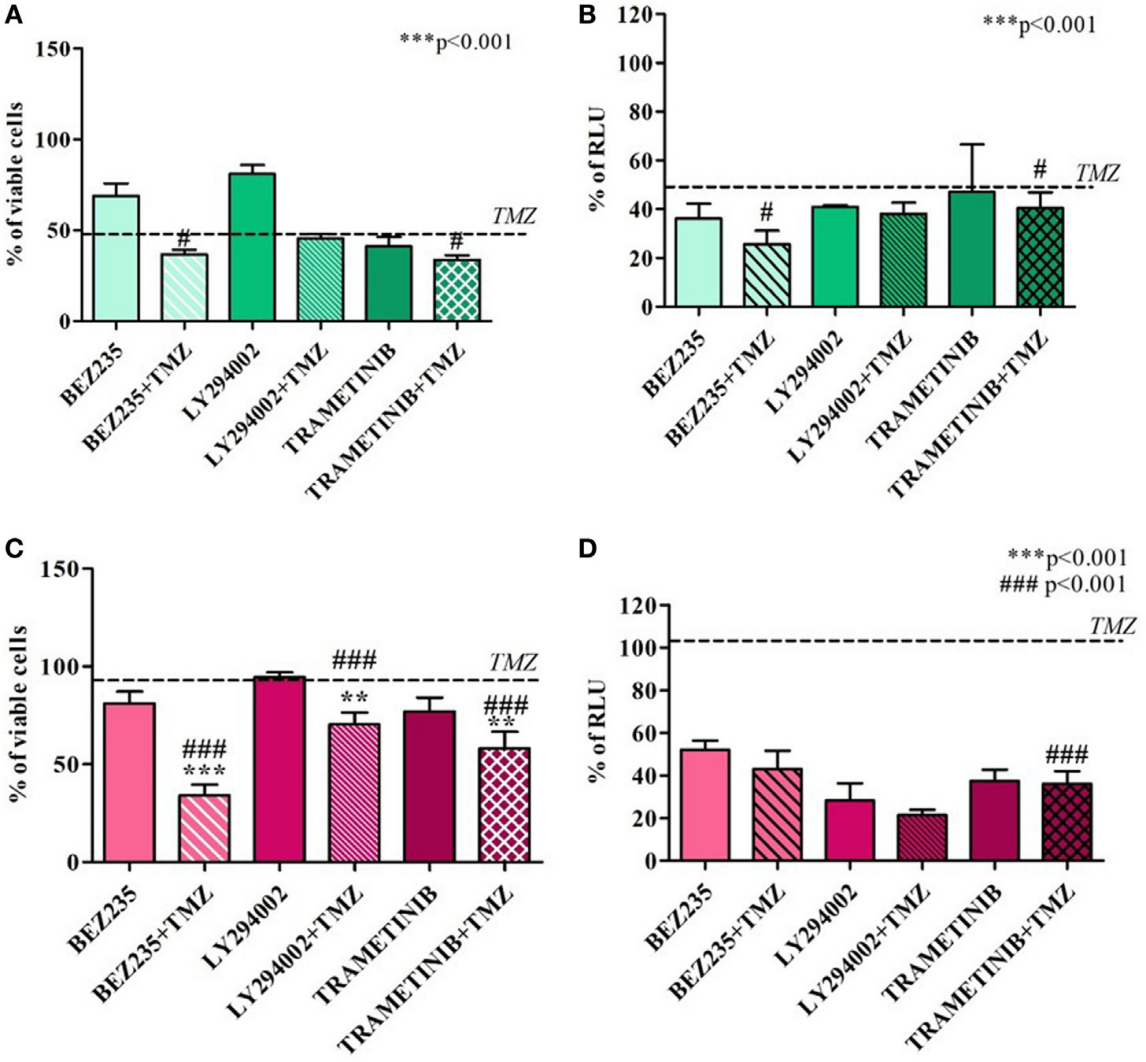
Cell viability and hypoxia-inducible factor (HIF)-1α activity after treatment with different HIF-1α inhibitors and temozolomide (TMZ). The viability of U251 **(A)** and T98 **(C)** was assessed by means of Trypan blue exclusion test and expressed as the percentage of viable cells after HIF-1α inhibitors. For U251 cells, all treatments are statistically significant vs control cells ***p < 0.001; ^#^*p* < 0.05; ^###^*p* < 0.001 HIF-1α inhibitors vs TMZ treatment. For T98, **p < 0.01; ***p < 0.001 treated vs control cells; ^###^*p* < 0.001 treatments vs TMZ treatment. HIF-dependent luciferase activity was analyzed in cell lysates, and expressed as the percentage variation in relative luminescence units (RLUs) in U251 **(B)** and T98 **(D)** cells. All treatments are statistically significant vs control cells ***p < 0.001; ^#^*p* < 0.05; ^##^*p* < 0.01; ^###^*p* < 0.001 HIF-1α inhibitors vs TMZ treatment.

Both in U251 and T98 cells, as expected, every treatment produced a statistically significant reduction in HIF-1α activity. In U251 TMZ-responsive cells, cell viability was impaired by all of the inhibitors. Moreover, although the high responsiveness to TMZ, comcomitant treatment with BEZ235 and Trametinib, produced a further decrease in cell viability after 24h (Figures 4A,B).

In T98 TMZ-resistant cells, as already reported after HIF-1α silencing, only concomitant treatments with TMZ produced a significant reduction in cell viability (Figures 4C,D).

### HIF-1α Persistence Induced by Blocking CMA Reverts TMZ Responsiveness in Sensitive Cells

**Figure 5:**
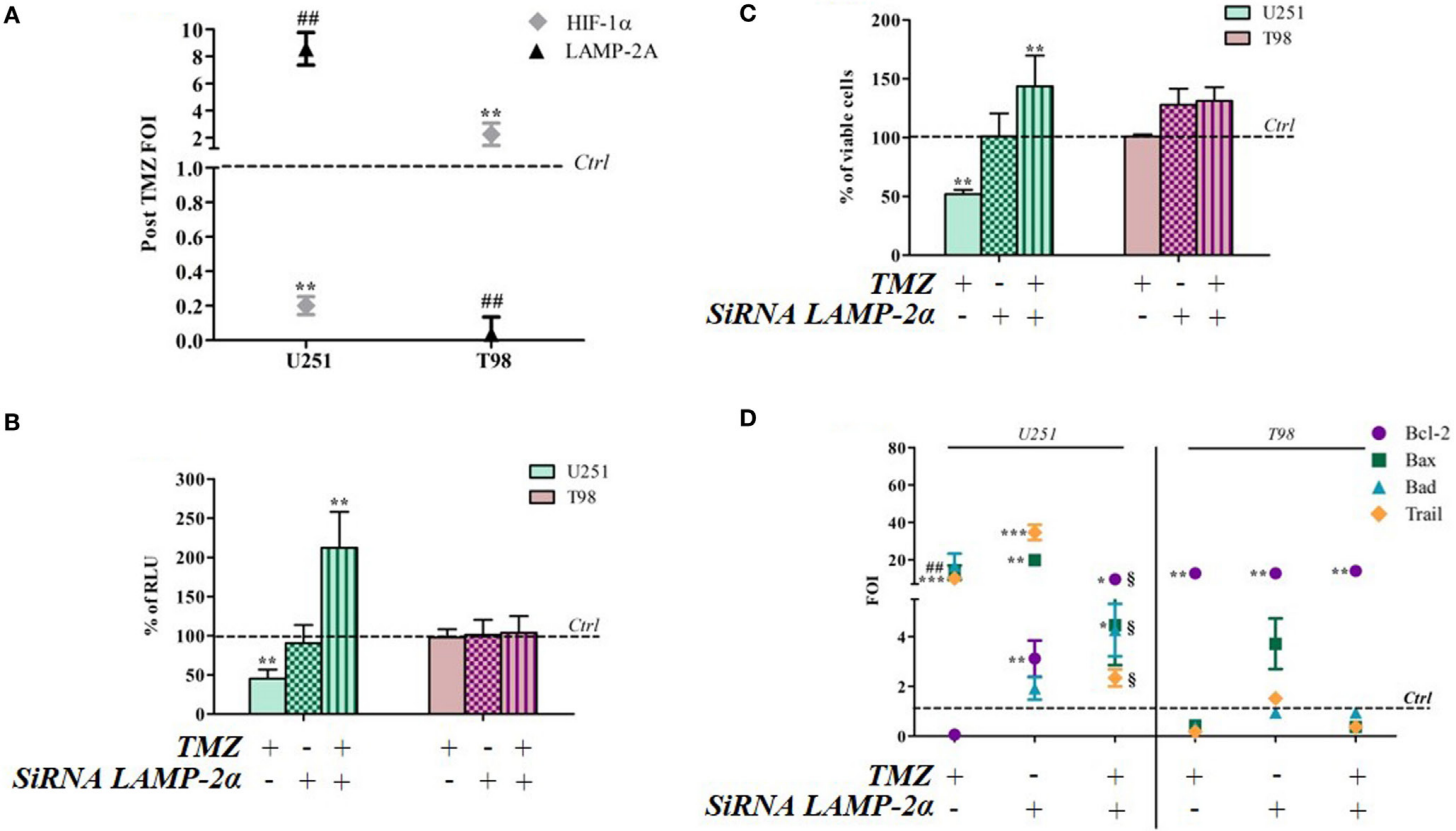
LAMP-2A silencing induces a resistant-profile in temozolomide (TMZ)-sensitive cells. **(A)** The expression of hypoxia-inducible factor (HIF)-1α and LAMP-2A was measured in the U251 and T98 cell lines after TMZ treatment. The data were normalized to β-actin, and the ΔΔCt values were expressed as folds of induction (FOI). **(B)** After silencing, the cells were treated with TMZ and the induction of *Bax, Bad, Trail*, and *Bcl-2* genes was analyzed by means of real-time PCR. The data were normalized to β-actin, and the ΔΔCt values were expressed as FOI of the ratio between treated and untreated cells. *p < 0.05; **p < 0.01; ***p < 0.001 treated vs control cells; ^#^*p* < 0.05; ^##^*p* < 0.01 siRNA LAMP-2A vs TMZ. ^1^*p* < 0.05 siRNA LAMP-2A + TMZ vs TMZ. **(C)** The viability of U251 and T98 cells was assessed by means of Trypan blue exclusion test, and expressed as the percentage of viable cells after single or combined treatment **p < 0.01 treated vs control cells. **(D)** HIF-1α-dependent luciferase activity was analyzed in cell lysates and expressed as the percentage change in relative luminescence units (RLUs) **p < 0.01 treated vs control cells. Mean values ± SD of three independent experiments.

Hypoxia-inducible factor-1α can be degraded by various mechanisms, one of which is the CMA machinery whose main player is LAMP-2A. TMZ treatment induced a reciprocal ratio of HIF-1α and LAMP-2A expression levels in TMZ-sensitive and TMZ-resistant cells (Figure 5A), whereas no difference was found in the same cell lines under control conditions (Figure S4 in Supplementary Material). With the aim of investigating the role of CMA in TMZ responsiveness, LAMP-2A was silenced, and the effect of TMZ was monitored by assessing cell viability, HIF-1α activity, and the modulation of apoptosis-related genes (silencing efficiency is shown in Figure S5 in Supplementary Material). LAMP-2A silencing alone did not induce any significant change in cell viability or HIF-1α activity in both TMZ-sensitive and TMZ-resistant cell lines (Figures 5B,C). However, LAMP-2A silencing in TMZ-treated sensitive cells was accompanied by the acquisition of resistant-like behavior characterized by increased cell survival after treatment and increased HIF-1α activity. This effect was not observed in the resistant cells.

LAMP-2A downmodulation did not change the expression profile of TMZ-resistant T98 cells after TMZ treatment, but LAMP-2A silencing induced a profound change in the expression of apoptosis-related genes in TMZ-treated U251 cells that led to a “resistant-like pattern” and confirmed the results of the viability assay (Figure 5D).

## Discussion

This study investigated the role played by HIF-1α activity in the susceptibility of GBMs to TMZ treatment, and its role as an early biomarker of tumor response to this drug and has led to us proposing that CMA is the mechanism responsible for modulating such activity.

MGMT methylation has become the main prognostic factor in GBM patient management (8, 34), but some cases have been reported in which MGMT-methylated cells are resistant to TMZ and MGMT-expressing cells are sensitive to the drug. This variability suggests that other, still unknown mechanisms, may be involved in TMZ resistance/responsiveness. Suggested GBM genetic biomarkers of the efficacy of TMZ (35) include isocitrate dehydrogenase 1 and 2 (*IDH1/2*) gene mutations and 1p and 19q chromosomal co-deletions (36, 37) which have acquired clinical relevance because of their diagnostic, prognostic, and sometimes predictive value even though they are still related to the status of genome methylation (9, 11, 12). There is therefore a need to identify new and early biomarkers capable of estimating the responsiveness of GBMs to TMZ that take into account other aspects of the tumors.

With the aim of identifying the cellular mechanisms involved in responses to TMZ, we analyzed four different human GBM cell lines and found that TMZ concentrations capable of inducing a statistically significant reduction in cell viability increase under hypoxic conditions. These data, together with previous findings by Lo Dico et al. (13) showing that a significant reduction in HIF-1α activity precedes a response to TMZ treatment in GBM cells, led us to explore this activity further, and we found that changes in HIF-1α activity correlate with TMZ-induced changes in the viability of TMZ-sensitive and TMZ-resistant cells under both normoxic and hypoxic conditions. The modulation of HIF-1α activity after TMZ treatment in all of the cell lines correlated HIF-1α nuclear translocation and the expression and release of its direct target VEGF, thus confirming previous results and the important consequences of this modulation on responsiveness. Our assessment of apoptosis-related gene balance after TMZ treatment under both normoxic and hypoxic conditions allowed us to delineate a reciprocal expression pattern in TMZ-sensitive and TMZ-resistant cells. The fact that hypoxia induced an expression pattern similar to that induced by TMZ treatment in resistant cells, in both cell types, provides a further clue concerning the importance of HIF-1α activity in GBM responsiveness to TMZ.

The HIF family not only includes the well-known HIF-1α and HIF-1β proteins but also other members such as HIF-2α, which has attracted a certain interest as it is induced by chronic hypoxia, and is involved in the regulation of metabolism and angiogenesis (38–40), and the maintenance of the stem niche, which suggests it is related to an aggressive profile (41). Our current findings demonstrate that HIF-1α (but not HIF-2α) expression can be modulated by TMZ treatment and that only HIF-1α modulation is responsible for GBM responsiveness to the drug. This thesis is strengthened by HIF-1α silencing experiments in resistant cells post-TMZ treatment: cell viability assay show that HIF-1α downmodulation alone is a necessary and sufficient condition for restoring drug responsiveness. The TMZ treatment of resistant cells in which HIF-1α is silenced, increases the transcription of pro-apoptotic genes, and concomitantly decreases *Bcl-2* expression, thus re-establishing a “sensitive-like” gene expression pattern.

Hypoxia-inducible factor-1α degradation through CMA is mainly due to LAMP-2A activity (30). As TMZ induces autophagy and HIF-1α possesses the KFERQ-like target motif needed for CMA degradation (29, 30), we hypothesized (and have subsequently demonstrated) that inhibiting CMA by silencing LAMP-2A not only inhibits HIF-1α downmodulation in sensitive GBM cells but it is also correlates with their acquisition of “TMZ-resistant behavior.”

The consistency in the modulation of *Bcl-2* expression observed in relation to the efficacy of TMZ treatment and after the modulation of HIF-1α activity (HIF-1α or LAMP-2A silencing and TMZ treatment) provides evidence of the key role played by HIF-1α activity as a switch between cell survival and cell death after TMZ treatment (42, 43).

Emerging evidence has shown that HIF-1α activity plays a fundamental role in many different tumors as it is not only involved in cell responses to hypoxia but also in cell bioenergetic balance, the maintenance of stemness, and invasiveness (44–46). For example, it has been reported that the metastatic potential of breast cancer is increased in patients undergoing anti-angiogenic therapy, possibly because of positive modulation of HIF-1α activity under hypoxic conditions. These data are in line with the observation that 25–40% of invasive breast cancer samples are positive for hypoxia markers (47–49). HIF-1α is also an important prognostic indicator in clear cell renal carcinoma (50) and recent studies have shown that it plays a role in the pathophysiological evaluation of gliomas as HIF-1α silencing combined with radiation therapy increases the therapeutic efficacy of glioma treatment by regulating cell cycle- and apoptosis-related signaling pathways (51, 52).

In the same way, it has been previously demonstrated that there is a connection between the abrogation of HIF-1α activity and reduced tumor cell viability and migration, reducing consequently also the metastatic potential (45).

For this reason, a number of HIF-1α inhibitors have been used in combination with standard chemotherapy to treat melanomas (53), breast cancer (54), and GBMs (55) in clinical trials in which it has been found that HIF-1α inhibition increases tumor response to treatment and leads to a reduction of the tumor progression rate, thus making it an interesting new therapeutic option (24, 56–58). The same results have been obtained herein in U251 and T98 cells with different small molecules able to reduce HIF-1α activity. Further studies will be needed to understand which molecule is able to produce the highest synergy in combination with TMZ, minimizing off-target effects.

**Figure 6:**
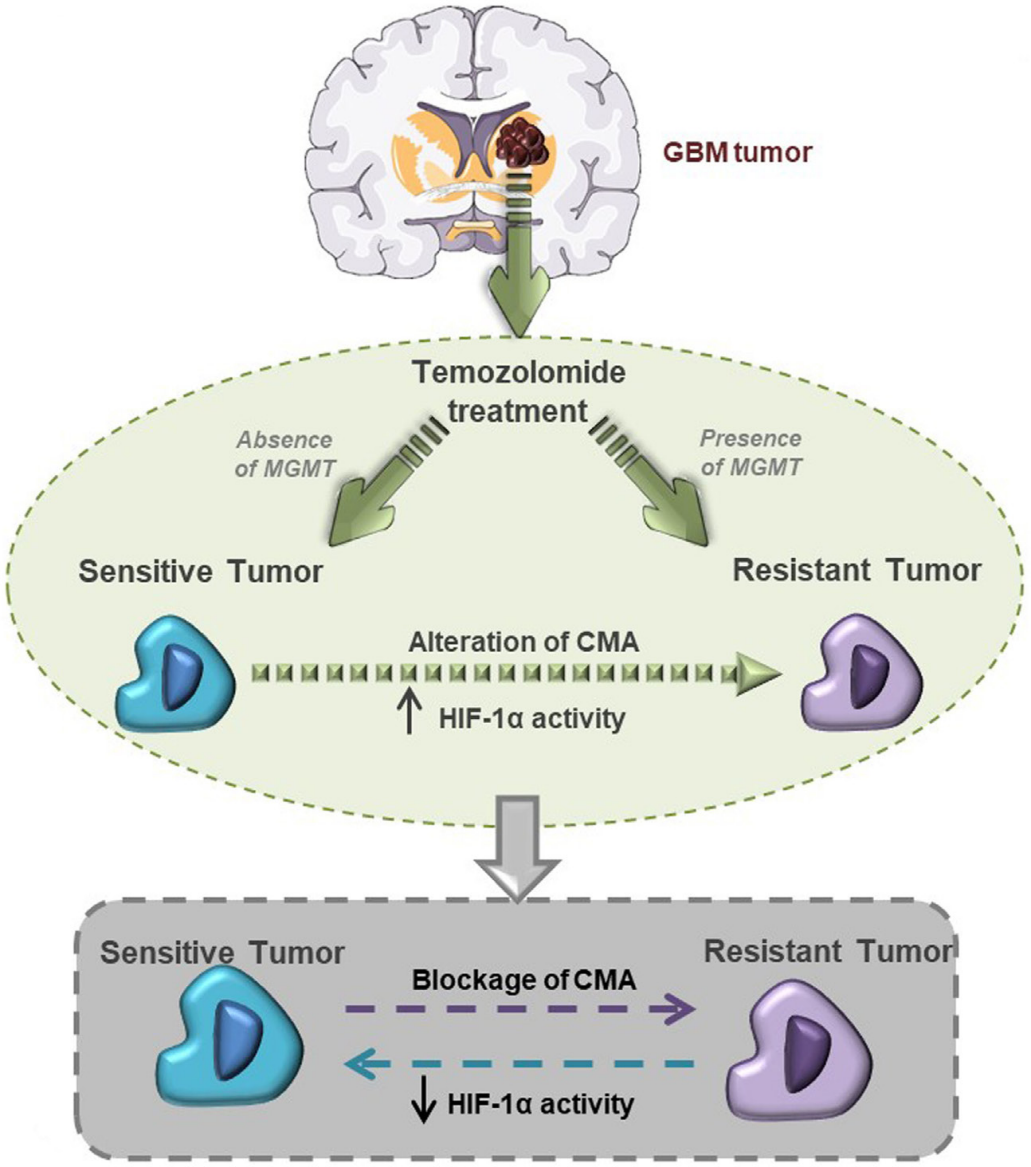
This graphical abstract explains hypoxia-inducible factor (HIF)-1α/chaperone-mediated autophagy (CMA) relation with glioblastoma responsiveness to temozolomide (TMZ).

In conclusion, our findings demonstrate the central role of HIF-1α activity in maintaining the TMZ resistance of GBM cells, and that CMA activity is the main mechanism mediating HIF-1α modulation and GBM responsiveness to TMZ (Figure 6).

## Materials and Methods

### Cell Lines and Reagents

The U251 cells were routinely maintained in RPMI 1640 medium, the U87 cells in Dulbecco’s modified Eagle’s medium, and the T98 and U138 cells in Eagle’s minimum essential medium. All of the media were supplemented with 10% heat-inactivated fetal bovine serum, penicillin and streptomycin (50 IU/mL), and 2 mM glutamine (all Euroclone, Italy). The cells were maintained in a humidified atmosphere of 5% CO_2_ at 37°C. For hypoxia experiments, all media were complemented with 25 mM HEPES Buffer to avoid media de-acidification in the absence of CO_2_.

Hypoxia-inducible factor-1α inhibitors were used as follow: 1 µM BEZ235 (for 48 h, SelleckChem, Houston, TX, USA), 50 µM LY294002 (Sigma-Aldrich), and 10 µM Trametinib (for 72 h, SelleckChem). All drugs were re-suspended in dimethyl sulfoxide in accordance with the manufacturer’s instructions.

The cell profiles are shown in Table 1.

**Table 1:** Glioma cell genetic profiles.

Cell line	Classification	MGMT methylation status (19)	PTEN	p53
U251	Glioblastoma (GBM)	Methylated	Mut	Mut
U87	GBM	Methylated	Mut	Wt
T98	GBM	Quite methylated	Mut	Mut
U138	GBM	Rarely methylated	Mut	Mut

### Cell Viability Assay

*In vitro* treatments were used to evaluate dose-dependent TMZ efficacy: 10,000 cells/cm^2^ were seeded in complete medium and treated for 24 h with TMZ 0.1–200 µM (Sigma-Aldrich, St. Louis, MO, USA), and cell viability was evaluated using the Trypan blue exclusion test. During the hypoxia experiments, 10,000 seeded cells/cm^2^ were incubated in a hypoxic chamber containing a 1% O_2_ gas mixture. In all the other experiments, the TMZ dose used was 100 µM.

### RNA Extraction and Real-Time PCR

RNA was extracted using a commercially available Illustra RNA spin Mini Isolation Kit (GE Healthcare, Milan, Italy) in accordance with the manufacturer’s instructions. Total RNA was reverse-transcribed to cDNA using a High-Capacity cDNA Reverse Transcription Kit (Applied Biosystems, Monza, Italy). The real-time PCRs were performed in triplicate for each data point using the Sybr Green technique; the oligonucleotides used are shown in Table 2. The changes in target mRNA content in relation to the β-actin housekeeping gene were determined using the ΔΔCt method.

**Table 2:** Primer sequences.

Gene	Forward	Reverse
BAX	5′-AGGGTGGCTGGGAAGGC-3′	3′-TGAGCGAGGCGGTGAGG-5′
BAD	5′-CCCAGAGTTTGAGCCGAGTG-3′	3′-CCCATCCCTTCGTCGTCCT-5′
HIF-1α	5′-TGATTGCATCTCCATCTCCTAC-3′	3′-GACTCAAAGCGACAGATAACACG-5′
HIF-2α	5′-CTTTTCGGGTCTGACAGCCT-3′	3′-TGTGTTCGCAGGAAGCTGAT-5′
VEGF	5′-CGAGGGCCTGGAGTGTGT-3′	3′-CGCATAATCTGCATGGTGATG-5′
TRAIL	5′-GCTCTGGGCCGCAAAAT-3′	3′-TGCAAGTTGCTCAGGAATGAA-5′
Bcl-2	5′-GATTGTGGCCTTCTTTGAG-3′	3′-CAAACTGAGCAGAGTCTTC-5′
LAMP-2A	5′-TGCTGGCTACCATGGGGCTG-3′	3′-GCAGCTGCCTGTGGAGTGAGT-5′
β-ACTIN	5′-ATCAAGATCATTGCTCCTCCTG-3′	3′-CTGCTTGCTGATCCACATCTG-5′

### HIF-1 Nuclear Quantification

An ELISA-based kit (TransAM Kit, Vinci-Biochem, Vinci, Italy) was used to detect and quantify HIF-1α transcriptional factor activity in accordance with the manufacturer’s instructions. The data are expressed as the amount of HIF-1α protein in nuclear extract (OD 450 nm).

### ELISA Assay

The concentration of VEGF in glioma cell-conditioned medium was quantified using an ELISA kit (VEGF Human ELISA KitNovex^®^, Cat. No. KHG011, Life Technologies, Monza, Italy) in accordance with the manufacturer’s protocol. The medium was collected under normoxic conditions and after hypoxia treatment also after TMZ treatment. The data are expressed in pg/mL.

### siRNA Transfection

Glioma cells were transfected with 10 nM of HIF-1α (Cat. No. GS3091, Qiagen, Milan, Italy) and/or EPASa/HIF-2α siRNA (Cat. No. GS2034, Qiagen) or a scrambled negative control (Product No. 1027280, Qiagen) using the Attractene Transfection Reagent (Cat. No. 301005, Qiagen) as indicated by the manufacturer. The cells used for LAMP-2A silencing were transfected with LAMP-2A siRNA or a scrambled negative control. LAMP-2A siRNA was custom made by Eurofins (Vimodrone, Italy).

### Bioluminescent Assay

For cell transfection, the glioma cell lines were seeded at 10,000 cells/cm^2^ and transfected with 1 µg of pHRE-luciferase plasmid. Twenty-four hours after transfection, the medium was collected, and the cells were processed for subsequent assay. The cells were analyzed using the GloMax-Multi Detection System (Promega, Milan, Italy). Protein content was measured by means of a Bradford assay, and the bioluminescence signal was normalized to milligrams of protein and expressed as relative luminescence units (RLUs = luciferase counts per milligram of proteins).

### Statistical Analysis

The *in vitro* experiments were repeated three times and led to reproducible results. The data are presented as the mean values ± SD of three independent experiments and were statistically analyzed using a *t* test or one- or two-way analysis of variance, followed by Dunnett’s or Bonferroni’s multiple comparison and Prism 4 software (GraphPad Software Inc., San Diego, CA, USA).

## Author Contributions

ALD: data generation, analysis and interpretation, and manuscript drafting. CM and CD: data generation, collection, assembly, and analysis. GL: critical review of the manuscript. LO: study conception and design, and critical review of the manuscript. All the authors approved the final version of the manuscript.

## Conflict of Interest Statement

The authors declare that they have no conflict of interest, and specify that no commercial company participated in or contributed to the preparation of the manuscript.
