# Supplementary material for: Hypoxia-Inducible Factor-1α Activity as a Switch for Glioblastoma Responsiveness to Temozolomide
Source: Front Oncol. 2018 Jul 2;8:249. doi: 10.3389/fonc.2018.00249 (PMC6036118; doi:10.3389/fonc.2018.00249)
Supplement: Supplementary file 1 [file Data_Sheet_1.PDF]

Supplementary

S.1

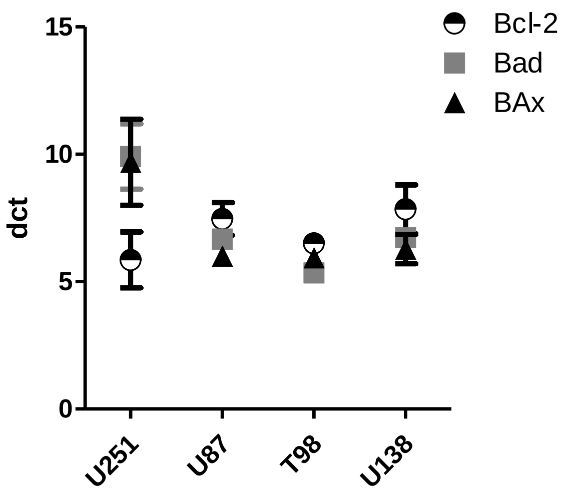

S.1 Basal expression of *Bcl-2, Bax* and *Bad* measured by Real time-PCR in all GBM cells. Data are expressed as Δct.

S.2

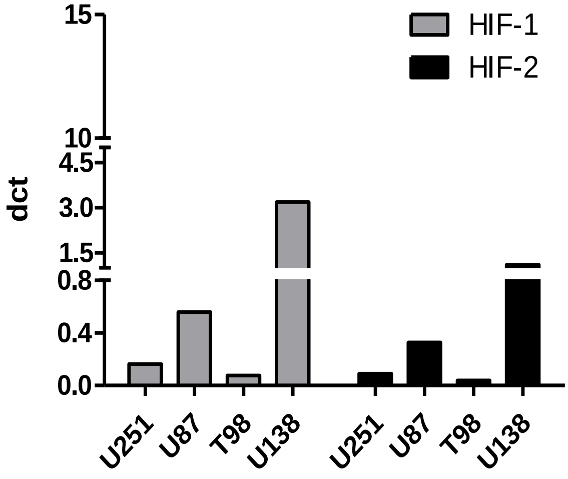

S.2. Basal expression of *HIF-1α* and *HIF-2α* measured by Real time-PCR in all GBM cells. Data are expressed as Δct.

Supplementary

S.3

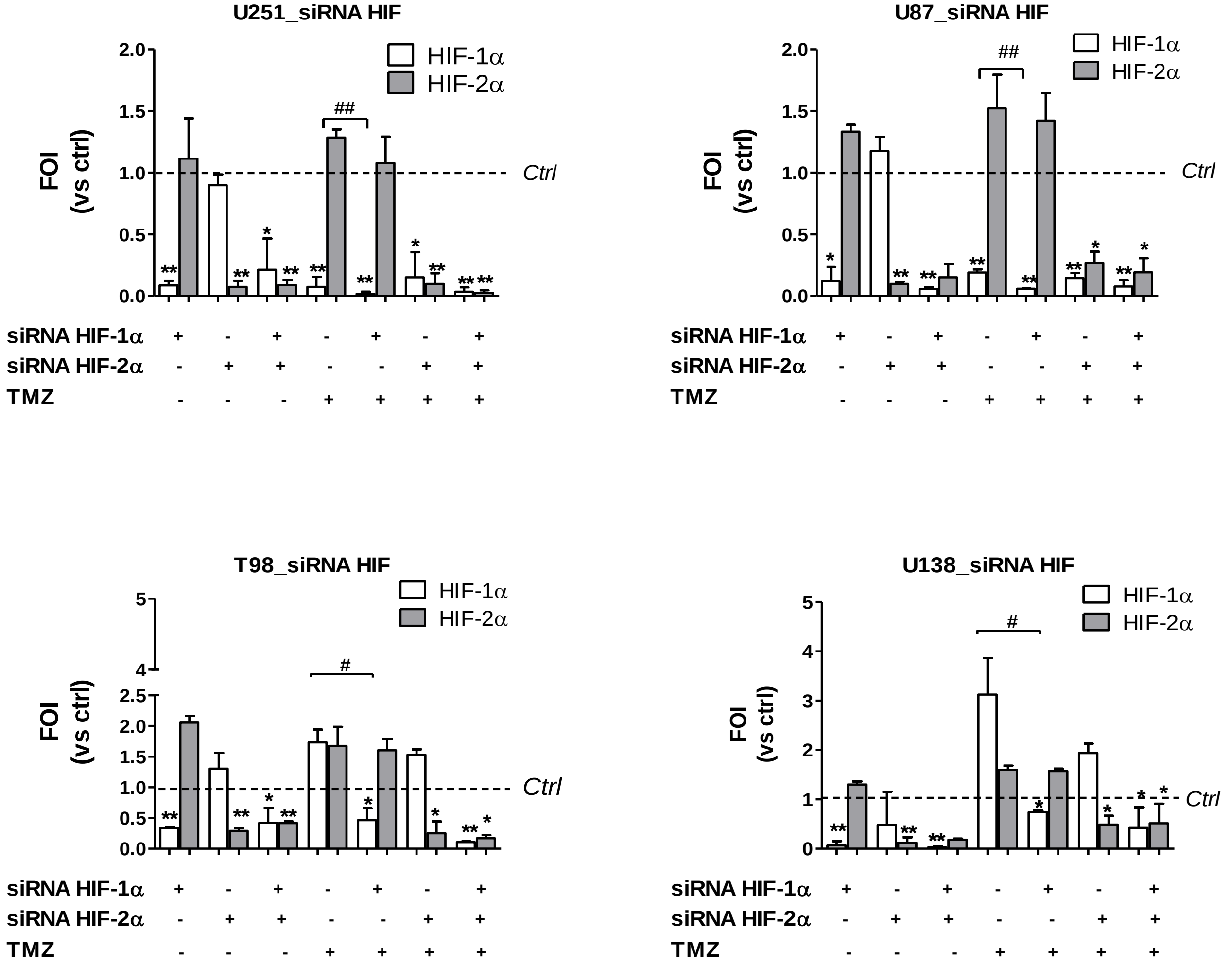

S.3. Real time-PCR for *HIF-1α* or *HIF-2α* after individual or concomitant HIF-1α/HIF-2α silencing in the presence or absence of TMZ treatment in U251 and U87 responsive cells (upper panel) and in T98 and U138 resistant cells (lower panel). Data were normalized for β-actin and ΔΔct values were expressed as FOI. * $p<0.05$; ** $p<0.01$ treated *vs* control (untreated cells). # $p<0.05$; ## $p<0.01$ siRNA+TMZ vs TMZ

Supplementary

S.4

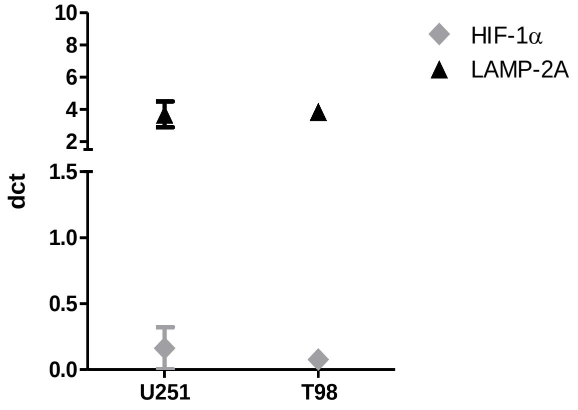

S.4. Basal expression of LAMP-2A and HIF-1α measured by Real time-PCR in U251 and T98 cells. Data are expressed as Δct.

Supplementary

S.5

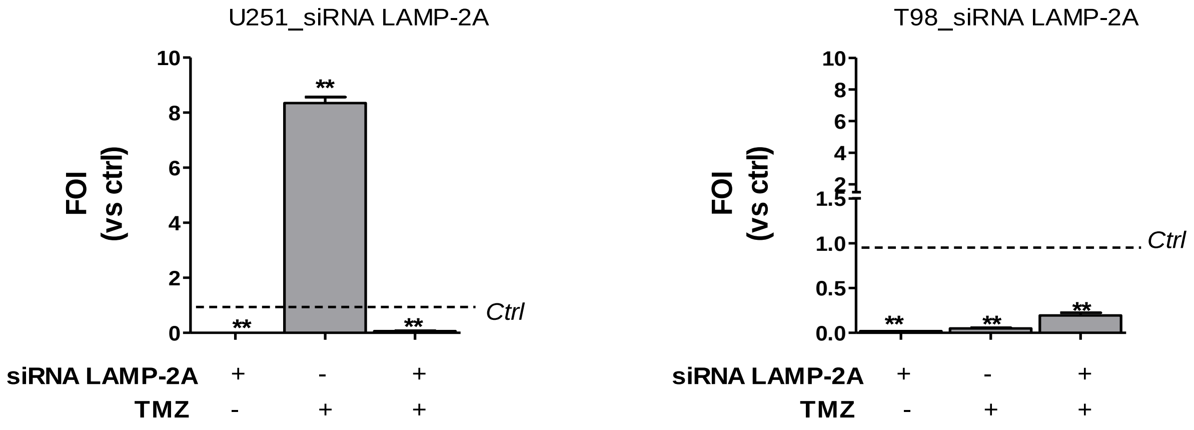

S.5. Real time-PCR for *LAMP-2A* after LAMP-2A silencing in the presence or absence of TMZ treatment in U251 and T98 cells. Data were normalized for β-actin and ΔΔct values were expressed as FOI. **$p<0.01$ treated sample *vs* control (untreated cells)
